# Social Factors Influencing Child Health in Ghana

**DOI:** 10.1371/journal.pone.0145401

**Published:** 2016-01-08

**Authors:** Emmanuel Quansah, Lilian Akorfa Ohene, Linda Norman, Michael Osei Mireku, Thomas K. Karikari

**Affiliations:** 1 Department of Molecular Biology and Biotechnology, School of Biological Science, University of Cape Coast, Cape Coast, Ghana; 2 Faculty of Health and Life Sciences, De Montfort University, Leicester LE1 9BH, United Kingdom; 3 Department of Community Health, School of Nursing, University of Ghana, Accra, Ghana; 4 School of Nursing and Midwifery, University of Health and Allied Sciences, Ho, Ghana; 5 Département Méthodes Quantitatives en Santé Publique (METIS), Ecole des Hautes Etudes en Santeé Publique, Rennes, France; 6 Ecole Doctorale Pierre Louis de Santeé Publique, Universiteé Pierre et Marie Curie (UPMC- Paris VI), Paris, France; 7 Meère et Enfant Face aux Infections Tropicales, Institut de Recherche pour le Deéveloppement (IRD), Paris, France; 8 Neuroscience, School of Life Sciences, University of Warwick, Coventry CV4 7AL, Uunited Kingdom; 9 Midlands Integrative Biosciences Training Partnership, University of Warwick, Coventry CV4 7AL, United Kingdom; The George Washington University School of Medicine and Health Sciences, UNITED STATES

## Abstract

**Objectives:**

Social factors have profound effects on health. Children are especially vulnerable to social influences, particularly in their early years. Adverse social exposures in childhood can lead to chronic disorders later in life. Here, we sought to identify and evaluate the impact of social factors on child health in Ghana. As Ghana is unlikely to achieve the Millennium Development Goals’ target of reducing child mortality by two-thirds between 1990 and 2015, we deemed it necessary to identify social determinants that might have contributed to the non-realisation of this goal.

**Methods:**

ScienceDirect, PubMed, MEDLINE via EBSCO and Google Scholar were searched for published articles reporting on the influence of social factors on child health in Ghana. After screening the 98 articles identified, 34 of them that met our inclusion criteria were selected for qualitative review.

**Results:**

Major social factors influencing child health in the country include maternal education, rural-urban disparities (place of residence), family income (wealth/poverty) and high dependency (multiparousity). These factors are associated with child mortality, nutritional status of children, completion of immunisation programmes, health-seeking behaviour and hygiene practices.

**Conclusions:**

Several social factors influence child health outcomes in Ghana. Developing more effective responses to these social determinants would require sustainable efforts from all stakeholders including the Government, healthcare providers and families. We recommend the development of interventions that would support families through direct social support initiatives aimed at alleviating poverty and inequality, and indirect approaches targeted at eliminating the dependence of poor health outcomes on social factors. Importantly, the expansion of quality free education interventions to improve would-be-mother’s health knowledge is emphasised.

## Introduction

Social determinants of health include the conditions in which people are born, live, work and grow, as well as measures that are put in place to curb illness [1]. The distribution of money, social resources, economies and political power shape these conditions at the national, regional and local levels [2]. Although earlier studies focused mainly on investigating social class and family income, recent studies have broadened the boundaries of what constitutes social determinants of health [1,3]. Social class encompasses factors influencing health and extends beyond simple measures of occupation and income; it includes family wealth, health literacy, education, employment, degree of autonomy in one’s job and quality of housing [2]. Ethnicity is also regarded as a social determinant although emphasis is usually placed on substructures defined by race, culture, family structure and gender [4]. Additionally, social relationships influence health and are therefore included in social determinant frameworks through constructs such as social support networks, social cohesion and social exclusion [5]. Furthermore, aspects of the natural environment such as climate change and the quality of water, air and soil are sometimes classified as determinants of child health [2,3]. It has been reported, for example, that infection by helminth (a free-living organism in aquatic and terrestrial environments) during pregnancy could affect motor and cognitive development (due to poor nutrition) among one-year-old infants [6]. However, the prescription of antihelminthics and vitamins to pregnant women in such environments during antenatal care can help to reduce anaemia by increasing haemoglobin concentration through to delivery and improving motor functions among their children [7]. This approach might be a good way to fight against childhood motor neuron diseases including Werdnig-Hoffmann disease, which can impair motor development and muscle movement later in life [8].

Scientists have been unable to provide a simple biological reason why the life expectancy at birth for men in the Calton region of Glasgow, Scotland, is fifty-four years, whereas that of men in Lenzie, just a few kilometres away, is eighty-two years, and why infant mortality rate among babies born to Bolivian women with no education is more than 100 per 1000 births compared with the less than 40 per 1000 babies born to women with at least secondary school education in the same country [1,2]. Evidence suggests that disparities of this nature could be reduced by improving the social environments within which people live and work [9]. Moreover, the rapid increase in the prevalence of diseases such as obesity is widely believed to be driven primarily by changes in lifestyle patterns [1,3]. However, in spite of the global interest in equity and social justice, knowledge on the social determinants of health has not yet resulted in the expected policy changes it deserves [3].

With early life events known to exert strong influences on health status in childhood and beyond, many child health researchers now consider a wide range of early life exposures in research on social determinants: these include caregiving and quality of parenting, maternal depression, home organisation, exposure to domestic violence and neighbourhood safety [3]. Humans possess a great deal of plasticity during the early years of life, helping to ensure rapid responses to changing environmental factors. This also makes children particularly susceptible to both positive and negative exposures [3]. Thus, when exposed to adversity, some of the ensuing changes can be maladaptive, potentially leading to bigger problems in adulthood [10]. For instance, depressed mothers are less attentive and sensitive to their newborns, failing to appropriately respond to the babies’ emotional signals [11]. Some encephalography studies have shown that such infants do not only develop shorter attention spans due to decreased frontal cortex activity but they also record persistent elevated heart rates and cortisol levels, which re-programme their internal “set point” to stress, and increase their risk of developing hypertension and coronary artery disease later in life [11–13]. Consequently, seemingly harmless and avoidable risks such as maternal depression could disturb human development and exert deleterious effects on lifelong health [3]. As Ghana is unlikely to achieve the Millennium Development Goals’ target of reducing child mortality by two-thirds between 1990 and 2015 [14], it is necessary to identify social determinants that might have contributed to the non-realisation of this goal. This would help towards the achievement of current and future plans (such as the Ghana national newborn health strategy and action plan, which hopes to help reduce neonatal mortality from 3.2% in 2014 to 2.1% in 2018 [15]). Here, we examined the published literature in this area in order to identify social factors influencing child health in the country.

## Methodology

We systematically reviewed the available literature for studies investigating social factors and their influence on child health in Ghana. The review process conformed to guidelines outlined in the PRISMA (Preferred Reporting Items for Systematic Reviews and Meta-Analyses) statement [16].

### Eligibility Criteria, Data Sources and Search Strategy

**Fig 1:**
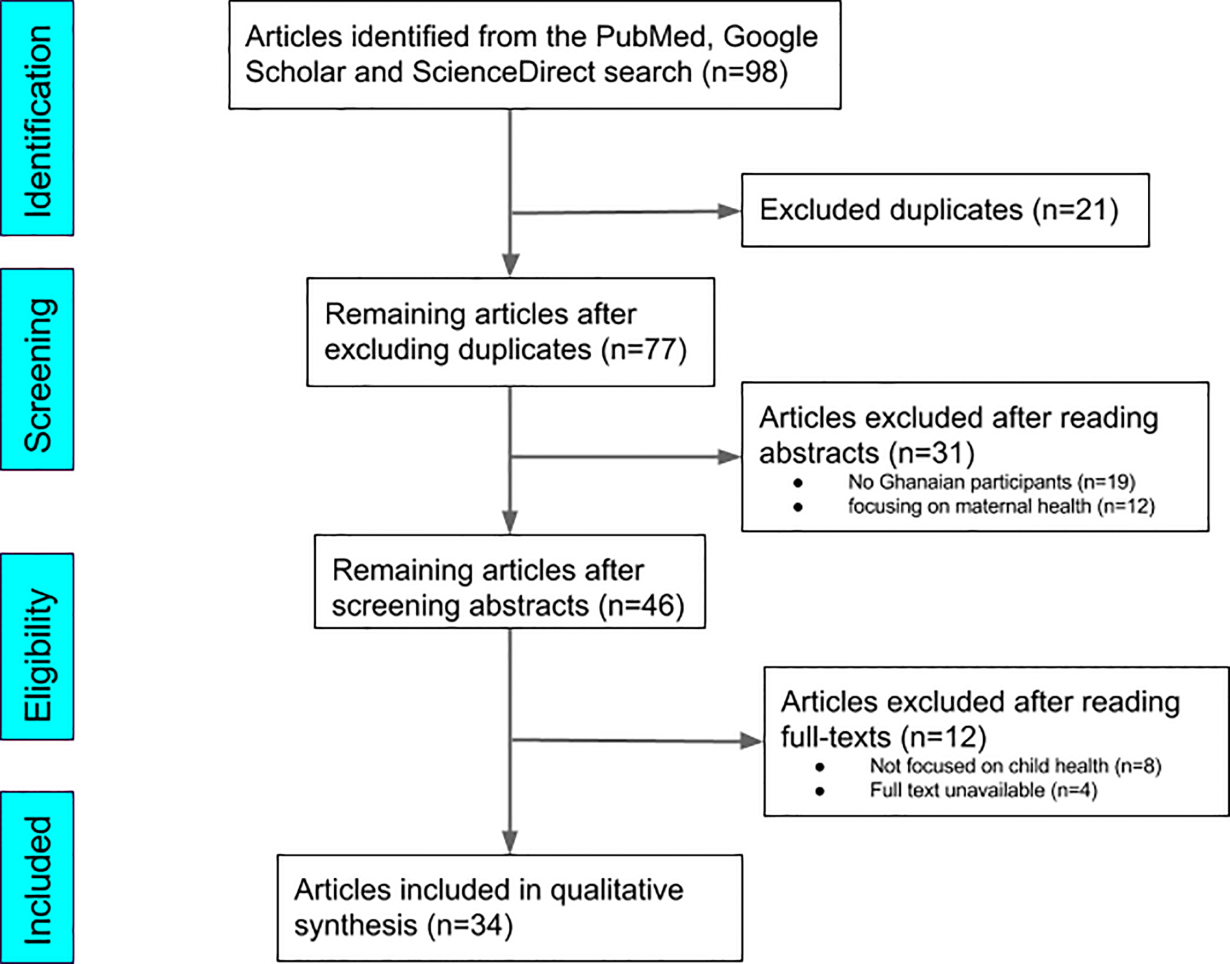
Flow diagram showing the article selection process, using the PRISMA approach.

Cross-sectional, retrospective and case-control studies on Ghanaian participants published in peer-reviewed journals were considered for inclusion. For the purposes of this systematic review, a “child” was defined as a person below the age of five years. ScienceDirect, PubMed, MEDLINE via EBSCO and Google Scholar were searched for published articles reporting on the influence of social factors on child health in Ghana. The search terms used included: “social determinants”, “child health”, “social factors influencing health” and “social determinants of child health” in combination with “Ghana”, “West Africa” or “sub-Saharan Africa”. As an example, the PubMed search strategy was: ((“social determinants of child health Ghana” [MeSH Terms] OR “child health” [All Fields] AND “social determinants Ghana” [All Fields]) OR (“child health” [All Fields] AND “social determinants” [All Fields] AND “Ghana” [All Fields]) OR (“social factors influencing health Ghana” [MeSH Terms]) OR (“social factors” [All Fields] AND “health” [All Fields] AND “Ghana” [All Fields]) OR (“infant” [MeSH Terms] OR “adolescent” OR “child” AND “West Africa” [All Fields])). The identified articles were evaluated using their titles, abstracts and full texts to select those that would fit our inclusion criteria (Fig 1). The references cited in the identified articles were also scanned for potentially useful articles that might have been missed during our search. The inclusion criteria were that the study must (i) have reported on social determinants of health on child health, (ii) have been conducted in Ghana or focused on Ghana, and (iii) have been indexed in the databases up until 30^th^ May, 2015. Apart from not meeting the inclusion criteria given above, studies were also excluded if they focussed entirely on discussing the health of individuals older than five years.

### Study Selection and Data Assessment

All articles meeting the criteria outlined above were selected for further analyses. Studies reporting on social determinants of health in Ghana but focused on maternal, paternal or general health rather than child health were excluded. No restrictions were made in terms of study design, although duplicates and studies conducted outside Ghana were excluded. Fig 1 illustrates the article-selection process which followed the PRISMA guidelines (S1 PRISMA Checklist). Information extracted included publication data, study settings, study design, sample characteristics, key findings and identified social determinants.

## Results and Discussion

### Study selection and characteristics, and findings from individual studies

**Fig 2:**
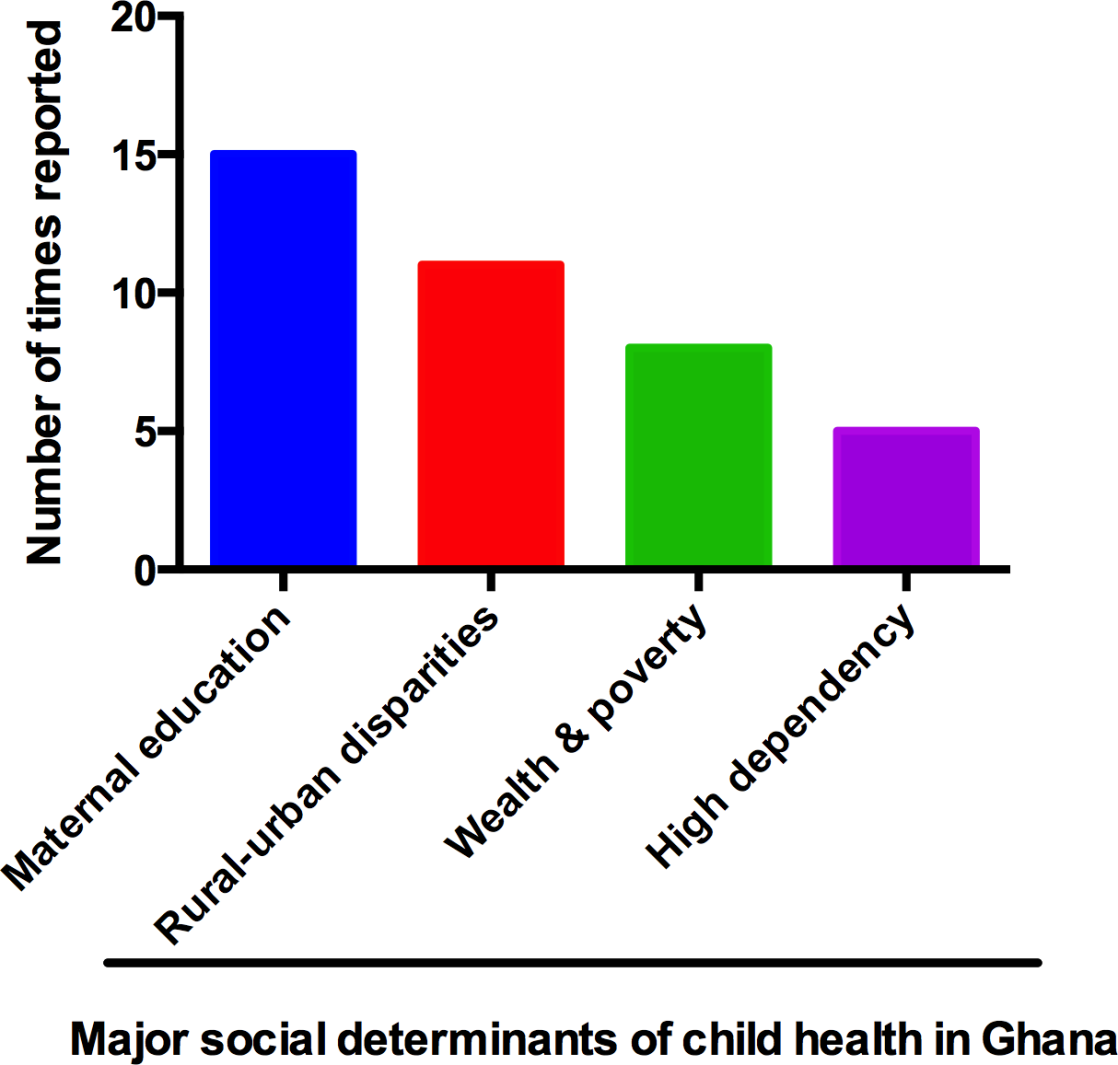
Major social determinants affecting child health in Ghana, ranked according to the number of times reported.

A total of 98 citations were obtained from the literature search. After removing entries that did not meet the inclusion criteria, the final selection included 34 publications. The major social determinants frequently reported included maternal education, rural-urban disparities, family income and high dependency (Tables 1 and 2). Other determinants of health identified were maternal age at birth, birth interval, father’s participation in childcare, alcohol and drug use by parents, childcare practices, cultural beliefs, child malnutrition, nucleation as well as training of healthcare personnel. We observed that most of these factors rarely acted in isolation; they rather usually acted in combination. With regards to this, the determinants identified in 27 articles were clustered into multiple interacting factors. Studies reporting on these aspects were published between 1988 and 2015. Twenty of these studies analysed data from nationally-representative samples, with the remaining examining data from various regions of the country, namely Eastern (2 studies), Northern (2 studies), Ashanti (3 studies), Greater Accra (2 studies), Volta (1 study), Central (1 study) and Upper East (1 study) regions. Additionally, one study was conducted among participants in the Volta and Eastern regions while another was done in the Ashanti and Eastern regions. The rest examined government policies. An intriguing aspect of the social determinants reported is that they appeared to be important for almost every disease studied. The health conditions associated with these factors included infant or child mortality, nutritional status of children, seeking treatment after burns, completion of immunisation programmes, health seeking and hygiene practices. The influence of each of the reported social determinants on child health has been discussed in Table 2 and Fig 2.

**Table 1:** Key findings of the publications included in this review.

Article	Setting	Study Design and Timeframe	Sample Characteristics	Key Findings	Identified Social Determinants
Brugha et al 1996 [17]	Eastern region	Structured interviews; 1991	294 mothers and 170 fathers having children aged 12–18 months (m) were interviewed	Father’s participation and high maternal education increased the likelihood of completing child immunisation programmes within 12 m	Father’s participation and high education enhanced the timely completion of child immunisation programmes
Hong 2007 [18]	Nationally representative sample	Structured interviews; 2003 GDHS	6,251 household interviews; 3,077 children (aged 0–59 m)	Children in the poorest 20% of households were more than twice likely to suffer from stunting compared to children in the richest 20%	Economic inequality (wealth and poverty) was a strong determinant of chronic childhood under-nutrition
Lavy et al 1996 [19]	Nationally representative sample	Household and community-level data from the second GLSS; 1988	Survey of children attending 231 health facilities	Increased availability of birth services, improved water and sanitation services and social infrastructure reduced child mortality rates	Eliminating rural-urban disparities would improve health status and decrease child mortality rates
Tolhurst et al 2008 [20]	Volta region	Focus group discussions, interviews, and Participatory Learning and Action methods; 2000–2004	Men (aged 18–80 y) and women (aged 18–77 y) were interviewed	Treatment-seeking behaviour for children was influenced by decision-making power, control over resources and the quality of relationships between elders, mothers and fathers	Gender transformatory approach, aimed at promoting women’s education and empowerment, can improve treatment-seeking behaviour for children
Addai 2000 [21]	Nationally representative sample	GDHS; 1990–1993;	Mothers visiting health facilities were interviewed	The use of MCH services were largely affected by level of education, religious background, place of residence, and to a smaller extent ethnicity and occupation	Improvement of MCH services requires changes in maternal education, rural-urban disparities and child care practices
Ahorlu et al 2006 [22]	Rural areas in southern Ghana	Structured interviews	Children under 5 y with malaria-related illnesses and 100 caretakers of children with malaria were interviewed	Only 11% of children with malaria-related illnesses received timely and appropriate treatments within 24 hours (h), and 33% within 48 h	Perceived risk and danger signs determine child health-treatment seeking behaviour more than economic, geographical and health access barriers
Allotey and Reidpath 2001 [23]	Selected communities in northeast Ghana	Qualitative study; structured interviews and focus groups	262 pregnant women were recruited at 28 weeks of gestation and 245 followed up until six weeks postpartum	Study of existing MCH data demonstrated that 15% of deaths of infants under three m of age were due to a belief in “chichuru” or spirit children, resulting in infanticide	Doing away with certain cultural beliefs may help in reducing child mortality
Ampaabeng and Tan 2013 [24]	Nationally representative sample	Data sources: GEIES data set 2003; GLSS II, 1988/89; GDHS, 1988	Cohort 1: aged 3–8 y, born between 1976 and 1981; cohort 2: aged 0–2 y born between 1981 and 1984	Negative impact of early childhood malnutrition on the cognitive development of famine survivors. The effects persisted into adulthood, resulting in poorer performance on cognitive achievement tests	Malnutrition affects the cognitive performance of children through to adulthood
Amugsi et al 2014 [25]	Urban and rural Ghana	Cross-sectional survey	1,187 dyads of mothers (aged 15–49 y) and their youngest child (aged 6–36 m)	Children with higher childcare practice scores had HAZ. Child’s and mother’s age, number of children <5 y, place of residence, wealth index were also significantly associated with HAZ	Associations exist between childcare practices, place of residence, wealth, dependency and child growth and health
Andrzejewski et al 2009 [26]	Central region	Data source: 2002 representative survey of communities and households in Ghana	2,500 participants in six districts of coastal Ghana	Even if a person is not literate, living in a community with high levels of literacy or a regular market still positively affected his/her health knowledge.	Social networks and diffusion in a community positively impacts health knowledge
Annim et al 2014 [27]	Selected communities in Ghana	Data source: last four rounds of the GDHS from 1993 to 2008	Participants included both males and females	Children under 5 y in nucleated households had better health outcomes than those in non-nucleated households	Nucleation, but not high dependency, positively impacted on child health
Antoine and Diouf 1988 [28]	Ghana, Benin, Kenya	Comparative study examining data collected within the period of 1977–1982; Data source: World Fertility Survey	Participants included both males and females	Infant mortality rates were lower in urban areas (in all countries) than in the rural areas; urban residents were mostly educated, and had regular sources of income	Urban residence, maternal education and high income positively affected child health outcomes
Armar-Klemesu et al 2000 [29]	Accra, Greater Accra region	Representative quantitative survey; January and March 1997	Survey involved 556 households with children < 3 y of age	Household socioeconomic factors were associated with preventive health seeking and hygiene behaviours	Poor maternal education was the main constraint for child feeding, health seeking and hygiene practices
Asenso-Okyere et al 1997 [30]	Nationally representative sample	GLSS round 1 1987/1988	3,200 households in 200 enumeration areas; 15,648 individuals	A positive correlation existed between mothers’ education and the nutrition level of children (aged < 5 y)	High maternal education was positively correlated with high nutritional status of children
Benefo 1995 [31]	Ghana, Ivory Coast, Cameroon	Cross-sectional survey data from the late 1970s	Survey involved several women who had children	Modernisation and female status was associated with declines in postpartum sexual abstinence which decreased maternal and child health	Declines in postpartum sexual abstinence decreased child health
Benefo and Schultz 1996 [32]	Ghana, Ivory Coast	GLSS1988/1989	3,200 households in 200 enumeration areas; 15,648 individuals	Household assets, maternal education and food prices impacted on child mortality in Ghana. Sanitation affected child survival only for mothers of low education levels	Maternal education, household assets (wealth), and food prices were strongly related with child mortality
Binka et al 1995 [33]	Northern region	Population-based case-control study	317 cases (infant and child deaths), and controls (living matched age, sex and locality); mothers of each case and control were interviewed	Risk factors for child mortality included delivery performed by untrained person, < 24 m interval between births, abuse of the child’s mother by the father, and the use of unprotected water source	How trained a birth attendant is, birth interval, abuse of mothers and unprotected water source were factors that strongly influenced child mortality
Brugha and Kevany 1995 [34]	Eastern region	Structured interviews;1991	Parents of 294 children	Completion of immunisation by year one was positively associated with town of residence (whether rural or urban), mother’s education, child's mother having < 5 children	Completion of immunisation was associated with maternal education, rural-urban disparity and dependency
Forjuoh et al 1995 [35]	Ashanti region	Community based survey	Survey involved children 0–5 y olds and their mothers in 50 enumeration areas	48% of children with burns were taken to health facilities; children in rural areas, those given first aid and those with flame burns were less likely to be taken to the hospital	Rural-urban disparity, administration of first aid and seriousness of burn were associated with likelihood for children to receive care at a health facility
Fosu 1992 [36]	Ghana, Zimbabwe, Kenya, Uganda	GDHS data; 1988–1989	4,201 to 7,150 participants in each country	20% and 30% of children with respiratory problems and fever respectively were treated. Mothers were afraid that injecting their children would lead to paralysis	Maternal education influenced immunisation of their children
Garg and Morduch 1998 [37]	Nationally representative sample	GLSS 1988/1989; cross-sectional	3,200 households (in 200 enumeration areas), involving 15,648 individuals	Children with only sisters as siblings did 25–40% better at health indicators than children having only brothers as siblings	Child health indicators were positively associated with having only sisters as siblings
Gram et al 2014 [38]	Nationally representative sample	Secondary analysis of vaccination card data collected on babies; 2008–2010	20,251 babies had 6 weeks follow-up; 16,652 had 26 w follow-up, and 5,568 had 1 y follow-up	Immunisation was delayed for: 27% of urban children, 31% of rural children, 21% of the wealthiest quintile, 41% of the poorest quintile, 9% of most educated group, and 39% of the least educated group	R/U disparity, wealth and maternal education significantly affected timeliness of child immunisation
Gyimah 2007 [39]	Nationally representative sample	GDHS round III and IV 1998 and 2003; cross-sectional	The 1998 survey included 4,843 women with 3,298 children; the 2003 survey included 5,691 women with 3,844 children	Religious differences did not influence child survival after controlling for confounding factors	Religious differences did not significantly affect child survival
Issaka et al 2015 [40]	Nationally representative sample	GDHS 2008; cross-sectional	822 children aged 6–23 m	Complementary feeding was significantly lower in infants from illiterate mothers. Other factors with similar outcomes included household poverty, no postnatal check-ups, non-Christian mothers and cultural beliefs	Low maternal education, cultural beliefs and household poverty were negatively linked to complementary feeding of infants
Issaka-Tinorga 1989 [41]	Ghana	NA	Review of government policies to curb child mortality	Three new interventions were required: protection of family income via alternative employment; village-level organisation for development; increased training of health personnel	Improving household assets (wealth), removing rural-health disparities and training more health personnel can help to decrease child mortality
Kanmiki et al 2014 [42]	Upper East region	Cross-sectional baseline survey of the Ghana Essential Health Intervention Project (GEHIP); 2011	3,975 women aged 15–49 y who had ever given birth	Mothers with less likelihood for child deaths were: those with basic school education (45% less likely); those in monogamous marriage (22% less likely); those below 20 y (11% less likely); those who are still married (27% less likely)	Factors that significantly predicted under-five mortality included mothers’ education level, presence of co-wives, age and marital status
Kayode et al 2014 [43]	Nationally representative sample	GDHS 2003, 2008; cross-sectional	6,900 women, aged 15–49 years (level 1), nested within 412 communities (level 2)	Infants of multiple-gestation, inadequate birth spacing and low birth weight as well as those with grand multiparous mothers and not breastfed were more likely to die during neonatal life	Multiparous mothers, lack of breast-feeding, infants of multiple gestation, inadequate spacing and low birth weight were factors positively associated with child mortality
Matthews and Diamond 1997 [44]	Nationally representative sample	GDHS1988; cross-sectional	4,488 females aged 15–49 y; sub-sample of 943 co-resident spouses; 3,690 children aged under 5 y	Over 50% of children aged > 11m who had a health card were not vaccinated; the most important predictors were maternal education, region of residence, and prenatal care	Maternal education, place of residence and prenatal care were strongly associated with child immunisation status
Nakamura et al 2011 [45]	Nationally representative sample	GDHS 1988–2008; Maternal Health Survey 2007; cross-sectional	These surveys covered 4,406, 5,822, 6,003, 6,251, 10,858, and 11,778 households in 1988, 1993, 1998, 2003, 2007, and 2008 respectively	Birth interval, bed net use, maternal education (secondary/higher), and maternal age at birth (17+ y) were associated with under-five mortality.	Maternal education, maternal age at birth, bed net use, and birth interval were associated with child mortality
Owusu-Addo 2014 [46]	Ahafo-Ano North and South districts (Ashanti region)	Semi-structured individual interviews	25 participants: 18 care-givers, 4 community leaders and 3 programme implementers	Conditional cash transfer services (CCTs) improved child health through major pathways such as: improved child nutrition, health service utilisation, poverty reduction, improved education and emotional health and well-being	CCTs helped in improving child health by addressing social determinants of health such as nutrition, access to health care, child poverty and education
Ruel et al 1999 [47]	Accra	Representative survey of 475 households in Accra (using questionnaires and interviews); 1997	Participants included households with children under 3 y	Good care practices related to child feeding and use of preventive health services were a strong determinant of children’s HAZ and compensated for the negative effects of poverty and low maternal education	Better use of good care practices such as improved child feeding practices and use of preventive health care could reduce malnutrition
Van de Poel 2007 [48]	Nationally representative sample	GDHS 2003; cross-sectional	Information on 3,061 children	Socioeconomic inequality in malnutrition is mainly associated with poverty, healthcare service use and regional disparities	Poverty, maternal education, healthcare access, family planning and regional disparities influenced malnutrition
Wirth et al 2006 [49]	Ghana, Cambodia, Ethiopia, Kenya	GDHS; Multiple Indicator Cluster Surveys 1998; Cross-sectional	4,488 females aged 15–49 y; sub-sample of 943 co-resident spouses; 3,690 children aged under 5 y	Inequality in childhood mortality was associated with differences in education, dependency and place of residence; highly-educated women and urban dwellers had much lower child mortality.	Maternal education, dependency and place of residence had impacts on child mortality
Yarney et al 2015 [50]	Ashanti and Eastern regions	Focus group discussions, in-depth interviews and key informant interviews	Young boys and girls and care-givers were included in the study as well as some key informants in the catchment areas	Care of children orphaned by AIDS was dependent on the following socio-cultural factors: traditional rituals and norms like funeral rites, marriage, festivals, inheritance and puberty rites as well as excessive alcohol intake, tobacco and drug use, and stigma	Care of orphaned children was affected by traditional activities and beliefs and social factors like increased alcohol intake, tobacco and drug abuse

CCTs, conditional cash transfer services; GEIES, Ghana Education Impact Evaluation Survey; GLSS, Ghana Living Standard Survey; GDHS, Ghana demographic and health Survey; HAZ, height-for-age Z-scores; m, month; NA, not applicable; R, rural; UP, urban poor; UR, Urban-rich; MCH, maternal-child health; y, year; AIDS, Acquired Immune Deficiency Syndrome.

**Table 2:** Major and minor determinants influencing child health in Ghana.

Social Determinant Framework	Major Determinants (Reported in Five or More Studies)	Minor Determinants (Reported in less than Five Studies)
Social class	Maternal education (reported in 15 studies)	Mother’s age at delivery; birth spacing; father’s participation in the child’s immunisation programme; perceived risks or danger signs; bed net use; social factors–alcohol and drug use; child care practices; marital status or wife co-habiting with husband.
Social or community relationships and health facilities	Rural-urban disparities (reported in 11 studies)	Training of health personnel; use of hospital facilities; cultural beliefs
Family income and dependency	Wealth/poverty (reported in 8 studies) and high dependency/multiparousity (reported in 5 studies)	Malnutrition; nucleation

### Impact of the Identified Social Determinants on Child Health in Ghana

#### Maternal Education

Infants are often reliant on mothers for their interactions with the environment [3]. From 1988 till date, many studies have looked at how maternal education affects the wellbeing of children in Ghana (Fig 2). In 1988, it was reported that child mortality was sensitive to maternal education [28]. However, the rate of child mortality declined as maternal education increased [28]. Later studies confirmed this positive association between low maternal education and high child mortality rates [32,42,45,49].

The association between maternal education and child mortality is not unique to Ghana; similar reports have come from other parts of the world [51]. For example, surveys conducted in some sub-saharan African countries provided initial evidence of the importance of maternal education on child survival, after controlling for socioeconomic factors such as place of residence and parents’ occupation [52]. Based on data from ten developing countries, a later study confirmed the significance of both maternal and paternal education in improving child health [53]. It was estimated that the impact of parental education was more significant than the combined effect of access to health services and income [53]. Additionally, more recent studies have shown that the association between maternal education and child mortality remains strong in the developing world [54–56]. While maternal education has a strong positive impact on child survival [54], the exact mechanisms involved remain to be established. However, treatment-seeking behaviour of educated and non-educated mothers may provide important insights. Studies in some developing countries found particularly strong evidence for protective roles of maternal education, indicating that educated mothers were more autonomous in making child health decisions and were more likely to seek treatment from well-resourced health facilities [57,58]. Greenaway and colleagues argued that maternal education was strongly associated with health knowledge, helping to explain the association between maternal education and the use of health services [57]. In Ghana, the relationship between maternal education and health-seeking behaviour is no different. An investigation into the use of injection in treating childhood diseases reported limited interest in participating in immunisation programmes, partly due to mothers’ low educational status, informing their fear of a possible link between injections and subsequent paralysis [36]. Subsequent studies showed that high maternal education had a positive association with the successful completion of immunisation programmes [34,38,44]. Another study reported that low level of maternal education was a main constraint against child feeding, health seeking and hygiene practices [29]. In line with this, two independent reports showed that strong positive correlations existed between high maternal education and nutritional status or complementary feeding of infants and children [30,40]. Additionally, van de Poel *et al*. [48] showed that a positive relationship existed between low maternal education and malnutrition. Similarly, a review of nationally-representative data on mothers interviewed at health facilities in Ghana concluded that efforts to improve maternal and child health services would require the promotion of education for women [21]. Taken together, the level of maternal education may inform health knowledge, complementary feeding, and mother’s treatment seeking-behaviour for their children. These factors may underlie the strong impact of maternal education on child survival.

#### Rural-Urban Disparities

Studies from some developing nations have revealed a significant impact of place of residence on child health. For example, child mortality rates were relatively lower in urban areas in Brazil compared to rural areas [59]. Reports from Kenya also showed that child mortality rates were higher in rural and urban slums compared to urban areas [60]. In Ghana, rural-urban residence is a crucial factor in determining child health outcomes. Antoine and Diouf [28] suggested that infant mortality rates were relatively lower in urban areas in Ghana, Senegal, Kenya, Benin and Cameroon [28]. Lavy *et al*. [19] and Wirth *et al*. [49] in two independent studies corroborated this finding, suggesting that eliminating rural-urban disparities by increasing access to birth services and improving water and sanitation services in rural areas would help to improve child health status. Brugha and Kevany [34] also reported that the completion of immunisation programmes within a year was positively associated with place of residence, maternal education and dependency. Similarly, subsequent studies showed that place of residence, wealth and maternal education significantly affected timeliness of child immunisation in Ghana [38,44]. Furthermore, several other studies have suggested that rural-urban disparities may be associated with various child health conditions in the country. For instance, van de Poel *et al*. [48] showed that rural-urban disparities interact with other factors such as poverty and maternal education to influence child malnutrition. Rural-dwelling children suffering from burns and those given first aid at home were less likely to receive care at a health facility [35]. Furthermore, a recent report suggested that place of residence in addition to other factors such as childcare practices and wealth index were significantly associated with a child’s height-for-age-Z-score (HAZ) [25]. Overall, place of residence plays crucial roles not only in child mortality but also in their nutritional status and the completion of immunisation programmes. The seeming comparative advantage of urban residency over rural residency in terms of positive health outcomes may be due to the ready availability of health-influencing services such as water and sanitation infrastructure, good housing, and modern healthcare services in urban areas compared to rural areas [59]. Therefore, to reduce child mortality in Ghana will require extra focus on the provision of essential services in rural areas and urban slums to match those in urban settlements. Moreover, developing measures to retain health personnel posted to rural areas would be a good strategy to improve child health [41].

#### Family Income (Wealth and Poverty Index)

A major theme in child health research focuses on understanding the relationship between national income and child mortality in developing countries. One such study estimated that should a country with an infant mortality of 50 per 1000 live births gain a 10% increase in the gross domestic product per capita purchasing power parity, the infant mortality rate will decrease to 45 per 1000 live births [61]. This demonstrates that wealth/poverty influences child survival. A strong impact of wealth/poverty status on child survival has also been recorded in Ghana. For example, Amugsi et al. [25] showed that children from homes with high household income had high HAZ. Hong (2007) [18] in an earlier study had reported that children in the poorest 20% of households were more than twice as likely to suffer from stunting compared to those in the richest 20% of the 6,251 households interviewed. A strong positive correlation between poverty and malnutrition has also been reported [48]. Household income is additionally a determinant of child health outcomes and child mortality [28,32], as well as the timeliness of immunisation [38] and complementary feeding of infants [40]. Together, financial status seems to be a strong determinant of malnutrition, child growth dynamics, participation in and completion of immunisation programmes, and child mortality in Ghana.

#### High Dependency/Multiparousity

An area given relatively less research attention is the effect of family size, polygamy, and multiparousity on child mortality [42]. However, high dependency influences child health outcomes [42]. A few studies have focused on this. Amugsi *et al*. (2014) [25] reported that high dependency strongly influenced a child’s growth in Ghana, with children in households having numerous under five-year-olds recording lower HAZ. This implies that high dependency correlates negatively with HAZ [25]. Brugha and Kevany (1995) [34] also demonstrated that low dependency (a child’s mother having less than five children) positively correlated with completion of immunisation programmes. Additionally, Kayode *et al*. [43] reported that children of grand multiparous mothers who were less breastfed were more likely to die during neonatal life. Furthermore, inequality in childhood mortality was closely associated with the level of dependency and the nuclear family type [27,49]

### Policy Implications of the Identified Social Determinants

For health and social interventions to be more successful in addressing the impacts of the identified social determinants, these policies must be better targeted [2]. An effective approach would have to recognise that social determinants are usually clustered into multiple interacting factors. Hence, effective interventions should be comprehensive and integrated [3]. Importantly, population-based interventions might be more effective compared to individually-focused interventions [2,3]. Social determinants could be addressed either directly by implementing policies focused on reducing poverty, social inequality and discrimination, and promoting education or indirectly through the implementation of strategies aimed at removing the association between social risks and poor health outcomes [25,26]. The proposed direct approach would emphasise the fundamental values of social equity and fairness. In reality, a combination of the direct and indirect approaches might be preferrable. The increasing rate of child mortality and the growing influence of social factors on inequities in child health outcomes require that more fundamental changes be made in how the child health care system is structured, organised and financed in Ghana. Although a national health policy and its implementation strategy for children under five years were introduced to help reduce mortality among children within this age bracket to 4% by 2015 (in line with the Millennium Development Goal number four), this goal is unlikely to be achieved and the implementation period for the policy expires at the end of 2015 [62,63]. Possible reasons accounting for the lack of realisation of this goal should therefore be carefully sought and studied and measures to eliminate such factors incorporated in future plans. We therefore propose the following strategies to help reduce the negative impacts of social factors on children’s health in Ghana.

#### Expand Interventions to Improve Access to Education

The Free Compulsory Universal Basic Education (FCUBE) policy was introduced in Ghana in 1995 with the aim of providing universal basic (primary and junior high school) education in the country by 2005 [64]. While the FCUBE policy was unable to achieve its target, it made significant contributions towards the improvement of basic education in the country [64]. The recent introduction of the capitation grant and the school feeding programmes has also helped to remove some barriers that poor families faced regarding their children’s education, mainly through the elimination of tuition fees and the provision of meals during school hours. These initiatives have helped to improve access to, and affordability of, education [65]. Of particular interest is that there seems to be a limitation in the number of girls (who are potential mothers) benefitting from the current interventions, possibly due to the poor access to education in the most remote areas [66]. For nationwide improvement in maternal education (as a means to improve child health outcomes), further rural education interventions are encouraged. These may include the establishment of schools in communities lacking these facilities, resourcing existing schools with essential learning tools and materials, and the development of motivation packages to encourage trained teachers to accept posting to rural schools. Since high maternal education is negatively correlated with child mortality [51], expanding the FCUBE policy to include senior high schools might also be beneficial towards improving literacy rates and health knowledge of would-be-mothers.

#### Strengthen the Community-based Healthcare Programme

Quality child healthcare delivery is partly dependent on the equitable distribution and retention of skilled healthcare professionals. However, health workers in Ghana are concentrated in urban areas, leaving most rural facilities understaffed [67,68]. This rural-urban disparity has had significant impacts on child health outcomes [19]. Identifying and effectively addressing human resource distribution gaps through the revision of existing human resources policies are critical [15].

Ghana has made substantial progress in strengthening quality healthcare delivery in rural areas through the introduction of the Community-based Health Planning and Services (CHPS) initiative [69–71]. Trained community health workers provide basic preventive and curative community health services for communities with otherwise limited access to healthcare. Following an initial testing, the CHPS project scale-up was launched in 2000 and it currently covers all districts in Ghana [72]. However, the pace of scale-up within the districts has been rather slow, despite efforts such as the Ghana Essential Health Interventions Programme (GEHIP) launched in 2010 to help accelerate this process and expand the range of services provided [72]. Health centres and CHPS compounds are organised to provide primary care at the community level, while secondary and tertiary health services are provided by district, regional and teaching hospitals [73]. While the establishment of CHPS compounds has helped to improve access to healthcare [69], there are however some problems confronting this programme. These include (i) accessibility–communities that are extremely remote from CHPS compounds are not adequately served by these services, and (ii) inadequate logistics and pharmaceutical product allocation [71]. In tackling these challenges, stakeholders should periodically review the location of existing CHPS compounds in relation to human settlements and establish new compounds as appropriate.

#### Address the Healthcare Financing Bottlenecks

A major determinant of child health in Ghana is poverty [18,48]. To provide risk protection to poor households and improve equity in the provision of healthcare, the country has turned to social health insurance [74–76]. Ghana’s National Health Insurance Scheme (NHIS) was launched in 2004 with the aim of providing a pro-poor healthcare finance system. The NHIS is an implementation of the World Health Assembly’s resolution that urged member states to ensure financial protection to all citizens, particularly children and reproductive-aged women [75]. The implementation of the NHIS has recorded many success stories, such as helping to reduce out-of-pocket health expenditure, particularly for the aged [76,77]. Notwithstanding the significant impact of the NHIS, there are compelling evidence pointing to inequities in enrolment; the scheme is generally not reaching the poor, with enrolment from the poorest quintile in the country being lower than the richest [74,75,78]. In addition, the need to provide risk protection and reduce financial barriers to maternal services led to a fee-exemption policy for antenatal care [79]. However, the rich has benefitted more than the poor for whom the policy was primarily introduced [79,80]. Moreover, only the first postnatal visit is reimbursed, leaving parents to directly fund subsequent visits through out-of-pocket interventions [15]. To ultimately achieve equitable financial protection for all citizens, reduce health-related financial risks, and ultimately improve child health services, NHIS enrolment and financing strategies should be revised to better accommodate and/or guarantee pre-payment for the poorest and most vulnerable households.

#### Establish Parent-Professional Partnerships with an In-built Common Accountability Framework

Efforts by the Government alone may not be enough to improve child health and survival in Ghana [81]. Effective transformation of the current healthcare system would require the efforts of all stakeholders, particularly healthcare professionals and families [17]. Family members are first-hand witnesses to the actual impacts of social determinants on child health, and often have insights into which improvements would be the most beneficial for their local communities [82]. This knowledge can, therefore, be harnessed via a community-based participatory research approach and parent-professional collaborative initiatives to design and implement population-specific interventions [81]. Creation of a web-based social networking technology for local measurement of social risks impacting health outcomes of children would also provide a means of empowering parents to act on behalf of their children both as individuals and as community members [2,3]. Establishing a parent-professional relationship as a reciprocal partnership with common health targets could contribute to reforming the healthcare system to improve quality and reduce cost [82]. A potentially-viable approach here would be to build on the initial success of the CHPS programme to forge stronger relationships between families, communities and healthcare professionals towards improving child health outcomes especially at the rural community level [72]. Moreover, efforts to improve child health and survival might be enhanced by the development of a common accountability framework. Although the implementation of the Ghana national child health policy and child health strategy has helped in reducing child mortality to an appreciable extent [14], incorporation of an accountability framework might have helped to further reduce the mortality rates. The development of a more effective approach for the measurement of outcomes may help in aligning existing disparate programmes along a set of common goals which may encourage cross-sector collaborations. For example, the development of the *Every Child Matters Framework* has enabled the United Kingdom to achieve a common accountability framework [2]. The framework stipulates five outcomes, namely (i) be healthy (ii) achieve and enjoy (iii) stay safe (iv) make a contribution, and (v) achieve economic well-being [2]. The framework also provides sets of quality-of-care measures (satisfaction of parents who care for children with disabilities) and quality-of-life indicators (breastfeeding and obesity prevalence) [2]. Such shared accountability at the local or community level could be useful in facilitating cross-sector innovation and improvement efforts, which are essential if healthcare and other service providers are to join forces in addressing the fundamental causes of adversity and provide systemic kinds of support [83].

#### Raise Community Awareness and Map Populations Based on Social Risks

Some community beliefs, cultural practices and attitudes have detrimental impacts on child health in Ghana [23,84]. For example, some communities promote the exclusive use of (i) traditional medicine as first-line treatment for sick children, and (ii) untrained traditional birth attendants in childbirth services [85,86]. Some traditional neonatal illnesses are often classified as non-clinically treatable [87]. An example is that 15% of infant deaths were attributed to a belief in “chichuru” or spirit children [23]. These beliefs and practices can cause significant delays in child-treatment seeking. In order for child health-related knowledge to be better translated into action, there is the need to increase awareness beyond healthcare practitioners and researchers [1]. Families, community members and opinion leaders need to be made better aware of the prevailing social risks in their communities and the influence that these risks have on health outcomes [3]. This would require that more community education activities be conducted at the national, regional and local levels. Currently, the existing community engagement capacity to advocate for behavioural changeis inadequate, notwithstanding the inclusion of such measures in reproductive, child and maternal health policies [15]. Further measures are therefore required.

Geographic information system mapping tools would also be useful for stakeholders to monitor patterns of disease epidemiology and social risks across local populations. Notable examples of the usefulness of this approach can be found in Canada and Australia, where an Early Development Instrument was developed to address the need for a uniform methodology for assessing the level of child development in the first year of schooling [88]. This serves as a good prototype for such an approach in Ghana. Data obtained from such mapping exercises might show the impact of gradients in social risk, providing communities with the needed information to tackle social issues especially through intervention and preventive strategies.

## Limitations

Most of the studies included in this review analysed data from nationally-representative surveys with individual and household response rates of about 96% and 99% respectively [43]. Although recall bias in this type of data collection is usually low, and appropriate stastistical methods were often used [43], there could be a problem with unobserved confounders in the studies using such data. For example, in estimating the impact of social factors on child mortality, it is possible that some infants may have died so early after birth that no social factor may have necessarily contributed to their death in which case the estimated dependence of their death on specific social factors may have been over-estimated. Moreover, because only surviving mothers had the opportunity to be interviewed, there remains a possibility that the association between child health and social factors may have been under-reported. Another limitation is that this review excluded some promising studies that were published solely in the abstract form (for example, in conference proceedings) as well as publications that were not indexed in the selected databases. This implies that the actual impact of some of the identified social factors may have been over- or under-stated and that perhaps some potentially high-impact social factors on child health in Ghana may have been missed.

## Conclusion

The available evidence shows that the major determinants of health that affect child health in Ghana include maternal education, rural-urban disparities (place of residence), family income levels (wealth/poverty) and high dependency. An intriguing aspect of these social determinants is that they appear to be important for various kinds of diseases, ranging from obesity to autism. However, in Ghana, these social determinants are reported to be heavily linked to child mortality, nutritional status of children, completion of immunisation programmes, health seeking behaviour and hygienic practices. Although evidence on the influence of social determinants on the health of children has been documented over one and halif decades ago and some policies have been introduced to address these determinants, more policy changes and better implementation strategies are required. In designing a strategy to address this problem, stakeholders would have to recognise that social determinants are usually clustered into multiple interacting factors. We propose that developing a national child health policy agenda which supports families via both direct and indirect approaches would be crucial in addressing social determinants of health inequalities and improving child health outcomes in the country.

## Supporting Information

S1 PRISMA Checklist A checklist of PRISMA guidelines followed in this systematic review. (DOC)

## Abbreviations

AIDS: acquired immune deficiency syndrome
CHPS: Community-based Health Planning and Services
FCUBE: Free Compulsory Universal Basic Education
GDHS: Ghana Demographic and Health Survey
GEHIP: Ghana Essential Health Interventions Programme
GEIES: Ghana Education Impact Evaluation Survey
GLSS: Ghana Living Standard Survey
HAZ: Height-to-age-Z-score
PRISMA: Preferred Reporting Items for Systematic Reviews and Meta-Analyses
WHO: World Health Organization
